# Supplementary material for: Content-rich biological network constructed by mining PubMed abstracts
Source: BMC Bioinformatics. 2004 Oct 8;5:147. doi: 10.1186/1471-2105-5-147 (PMC528731; doi:10.1186/1471-2105-5-147)
Supplement: Additional File 2 — The original results of the above study (non-essential files are deleted to keep the file size under the limit set by BMC bioinformatics). [file 1471-2105-5-147-S2.bz2 › chilibotAdditionalFile2/dip10/164ID8702683E570/html/GHR_STAT5A.html]

 


 **GHR** and **STAT5A** 
  
Found 75 abstracts in PubMed, retrieved 10.  
 

 What does Google say? 
 PDF only 
| .edu only 

---

**Interactive relationship** (e.g. stimulation, inhibition, etc)

**Inhibitory relationship**- The signal transducer and activator of transcription 5  [ **STAT5A** ]  STAT5  [ **STAT5A** ]  mediated transcriptional activation in mutant  **GHR**   **GHR**  T51I expressing cells was significantly reduced as compared with wild type  **GHR**   **GHR**  wt expressing cells.  Ref: 12423626 Growth Horm IGF Res, 2002
**Stimulatory relationship**- We show here that endogenously produced GHBP  [ **GHR** ] , in contrast to exogenous GHBP  [ **GHR** ] , was able to enhance the STAT5  [ **STAT5A** ]  mediated transcriptional response to GH.  Ref: 12488452 J Biol Chem, 2003
**Neutral relationship**- Thus, the GHBP  [ **GHR** ]  exerts divergent effects on STAT5  [ **STAT5A** ]  mediated transcription depending on its cellular location.  Ref: 12488452 J Biol Chem, 2003

**Non-interactive relationship** (e.g. studied together, co-existance, homology, etc.)

- The effect of heterodimerization was studied by comparing activation of Janus kinase 2, signal transducer and activator of transcription STAT 1, STAT3, STAT5  [ **STAT5A** ] , and MAPK in Chinese hamster ovary cells stably transfected with chimeric genes encoding receptors consisting of cytosolic and transmembrane parts of oGHR and oPRLR, extracellular domains of human granulocyte and macrophage colony stimulating factor hGM CSF receptor alpha or beta, and cells transfected with the two forms alpha or beta of PRLR and  **GHR** .  Ref: 12865335 Endocrinology, 2003
- Interestingly, when the GHBP  [ **GHR** ]  was targeted constitutively to the nucleus by the addition of the nuclear localization sequence of the SV40 large T antigen, greater enhancement of STAT5  [ **STAT5A** ]  mediated transcription was obtained.  Ref: 12488452 J Biol Chem, 2003
- In C2C12  **GHR**  cells, we observed an increased activation of the Janus kinase 2 Jak2, signal transducers and activator of transcription 5 Stat5  [ **STAT5A** ] , mitogen activated protein kinase MAPK and protein kinase B Akt upon acute GH stimulation.  Ref: 12681449 Cell Signal, 2003
- The transcriptional enhancement did not require GH per se and was not specific to the GH receptor  [ **GHR** ] , since similar enhancement of STAT5  [ **STAT5A** ]  mediated transcription by nuclear localized GHBP  [ **GHR** ]  was obtained with specific ligand stimulation of both prolactin and erythropoietin receptors.  Ref: 12488452 J Biol Chem, 2003
- Upon GH stimulation of the  **GHR**  positive CMT U335 canine mammary tumor cell line, the transcription factors  **STAT5A**  and STAT5B become phosphorylated on their tyrosine residues, which is likely to reflect the significance of mammary GH in vivo.  Ref: 12431808 Mol Cell Endocrinol, 2002
- STAT5  [ **STAT5A** ] , STAT3,  **GHR** , Socs 1 3, and Cis phosphorylation and abundance were assessed by using immunoblots, EMSA, and or real time RT PCR.  Ref: 12519742 Am J Physiol Gastrointest Liver Physiol, 2003
